# Effects of protonation on the hydrolysis of triphosphate in vacuum and the implications for catalysis by nucleotide hydrolyzing enzymes

**DOI:** 10.1186/s12858-016-0068-7

**Published:** 2016-06-29

**Authors:** Farooq Ahmad Kiani, Stefan Fischer

**Affiliations:** 1 Computational Biochemistry, Interdisciplinary Center for Scientific Computing (IWR), Heidelberg University, Im Neuenheimer Feld 205, D-69120 Heidelberg, Germany; 2 Research Center for Modeling and Simulation (RCMS), National University of Sciences and Technology (NUST), Sector H-12, 44000 Islamabad, Pakistan

**Keywords:** ATP hydrolysis, ATPase, GTPase, Enzymatic mechanism, Concurrent and sequential mechanisms, Computational biochemistry

## Abstract

**Background:**

Nucleoside triphosphate (NTP) hydrolysis is a key reaction in biology. It involves breaking two very stable bonds (one P–O bond and one O–H bond of water), in either a concurrent or a sequential way. Here, we systematically examine how protonation of the triphosphate affects the mechanism of hydrolysis.

**Results:**

The hydrolysis reaction of methyl triphosphate in vacuum is computed with protons in various numbers and position on the three phosphate groups. Protonation is seen to have a strong catalytic effect, with the reaction mechanism depending highly on the protonation pattern.

**Conclusion:**

This dependence is apparently complicated, but is shown to obey a well-defined set of rules: Protonation of the α- and β-phosphate groups favors a sequential hydrolysis mechanism, whereas γ-protonation favors a concurrent mechanism, the two effects competing with each other in cases of simultaneous protonation. The rate-limiting step is always the breakup of the water molecule while it attacks the γ-phosphorus, and its barrier is lowered by γ-protonation. This step has significantly lower barriers in the sequential reactions, because the dissociated γ-metaphosphate intermediate (P_γ_O_3_^−^) is a much better target for water attack than the un-dissociated γ-phosphate (−P_γ_O_4_^2−^). The simple chemical logic behind these rules helps to better understand the catalytic strategy used by NTPase enzymes, as illustrated here for the catalytic pocket of myosin.

A set of rules was determined that describes how protonating the phosphate groups affects the hydrolysis mechanism of methyl triphosphate: Protonation of the α- and/or β- phosphate groups promotes a sequential mechanism in which P-O bond breaking precedes the breakup of the attacking water, whereas protonation of the γ-phosphate promotes a concurrent mechanism and lowers the rate-limiting barrier of water breakup. The role played by individual protein residues in the catalytic pocket of triphosphate hydrolysing enzymes can be assigned accordingly.

**Graphical abstract:**

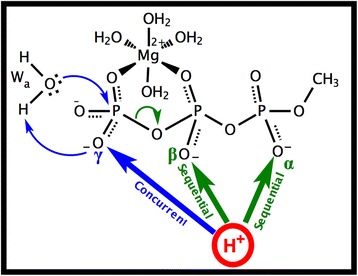

**Electronic supplementary material:**

The online version of this article (doi:10.1186/s12858-016-0068-7) contains supplementary material, which is available to authorized users.

## Background

**Fig. 1:**
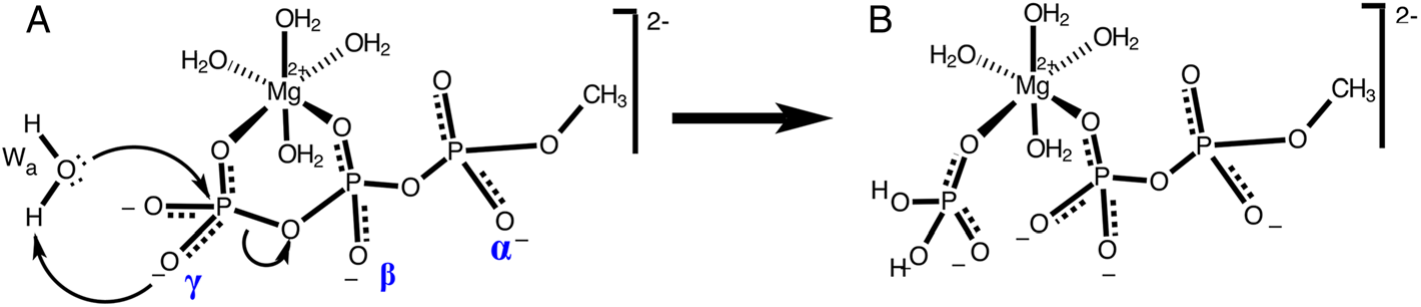
Triphosphate hydrolysis. **a** Unprotonated methyl triphosphate reactant. The three protonation sites (α, β, γ) are labeled. The *arrows* show the electronic rearrangements of the concurrent reaction mechanism: concerted breaking of the P_γ_–O_βγ_ bond and lysis of water W_a_. **b** Product state. The magnesium Mg^2+^ is hexa-coordinated by two oxygen atoms of the triphosphate and four water molecules

**Fig. 2:**
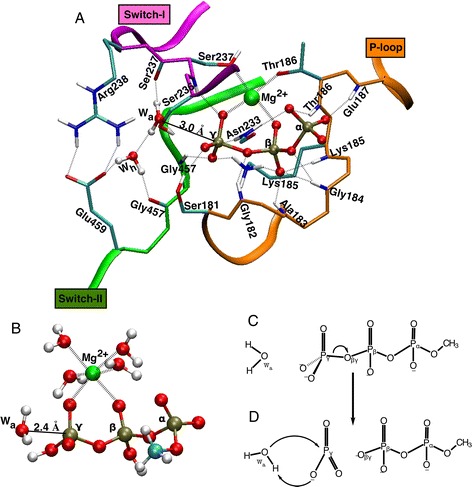
Reactant state structure of triphosphate. **a** ATP bound to myosin (based on the 1VOM crystal structure). Only the triphosphate moiety (labeled α,β,γ) of ATP is depicted. Two water molecules that coordinate Mg^2+^ are not shown. *Thin dotted lines* show hydrogen bonds shorter than 2.8 Å between the heavy atoms. **b** Methyl triphosphate (β- and γ-protonated), after energy minimization in vacuum. The Mg^2+^ hexa-coordination is depicted with dotted lines. **c** First step of the sequential mechanism: breaking of the P_γ_–O_βγ_ bond to form a stable P_γ_O_3_^−^ metaphosphate. **d** Second step of the sequential mechanism: lysis of water W_a_ and simultaneous attack of the metaphosphate

Nucleoside triphosphate (NTP) hydrolysis [1, 2] is an important enzymatic reaction in biology [3]. During this reaction, the triphosphate moiety is hydrolyzed into diphosphate and inorganic phosphate. The reaction requires the breaking of two stable bonds: the P_γ_–O_βγ_ bond of the triphosphate and one O-H bond of the attacking water molecule (W_a_ in Fig. 1). This makes NTPs highly stable in water [4]. For example for ATP in presence of magnesium at 70 °C and pH ~ 7, the hydrolysis reaction rate constant has been measured at a slow 4^.^10^−4^ min^-1^, which corresponds to a high free energy barrier of ~28 kcal mol^−1^ [5]. Two types of mechanisms have been proposed for the hydrolysis of triphosphate: [6, 7] 1) A concurrent mechanism, in which P_γ_–O_βγ_ cleavage and O–H bond breaking are concerted (Fig. 1). 2) A sequential mechanism, in which the P_γ_–O_βγ_ bond breaks (Fig. 2c) before the OH^−^ nucleophilic attack (Fig. 2d) [8, 9]. In vacuum, both mechanisms have similar high-energy barriers, respectively 44.0 and 45.9 kcal mol^−1^ for concurrent and sequential reactions. NTPase enzymes accelerate the hydrolysis reaction by a factor of 10^7^ [10]. To help understand the catalytic mechanism in NTPase enzymes, we are studying here the triphosphate substrate in vacuum, and investigate in particular how protonation of different phosphate groups affects the hydrolysis reaction.

In the catalytic pocket of NTPase enzymes, many partial positive charges surround the triphosphate moiety of NTP. Figure 2a shows, for example, the active site of myosin with ATP bound in the catalytically competent conformation. The many hydrogen bonds donated by the NH moieties of the P-loop (for “phosphate-binding” loop) to the triphosphate moiety are seen in all NTPase binding sites. The positive electrostatic environment is further enhanced by Lys185 and H-bond donor Asn233 (Fig. 2a). These lysine, [11–13] and aspargine [14] (replaced by an arginine in some NTPases) [12, 15–17] residues are critical for the catalytic function of NTPases, as evidenced from kinetic and mutagenesis studies. To differentiate how interactions between this electrostatically positive environment and the three individual phosphate groups affects the hydrolytic mechanism and its energy barrier, we are studying here the effect of placing protons on triphosphate in vacuum, varying the number of such protons and their oxygen location on the triphosphate. This reveals that the location of the protons has a strong influence on the catalytic mechanism. We derive a simple set of chemical rules, which explain this effect and can be related to the catalytic strategy used by NTPases.

Triphosphate tends to chelate Mg^2+^ in solution, and in the enzymes, NTPs are always found with one Mg^2+^ complexed between the β- and γ-phosphates. Therefore, it is important to study the triphosphate bound to Mg^2+^, [18] which should be fully coordinated. The pK_a_ value of the reaction [HATP^3-^ → ATP^4-^ + H^+^] is 6.95 at 25C^o^ in the absence of magnesium [19]. The presence of magnesium makes this pK_a_ drop by about 2 units (for example to 5.36 at 70C^o^) [5]. This means that the Mg/ATP complex is fully deprotonated in water, and therefore easily binds as Mg^2+^/ATP^4−^ to the enzymes. In the present study, only the triphosphate moiety of NTP is considered, using methyl triphosphate as a substrate analogue (Fig. 1, left panel). Five water molecules are present: one attacking (lytic) water W_a_ placed apically relative to the γ-phosphate, and four water molecules complete the coordination shell of the Mg^2+^. It can be seen in Fig. 2b that the energy-optimized conformation of this complex in vacuum is very similar to the conformation of the corresponding moiety of NTP in the enzymes (compare with Fig. 2a). Protonation states with up to three protons on the triphosphate were considered: A single proton was placed on either triphosphate oxygen α, β or γ (see Fig. 1a for atomic nomenclature). In the doubly protonated triphosphate, two protons were placed on either the αβ-, αγ-, or βγ-oxygens. In the triply protonated case, the three protons are on oxygens α, β and γ (named here “αβγ” protonation). Together with the un-protonated (zero protons) case, a total of eight protonation states were investigated here. For each of these protonation states, it was attempted to obtain both a concurrent and a sequential reaction. Using the AM1/d quantum method, (with parameters optimized for the treatment of phosphates chelating magnesium, see Methods), [20] minimum energy paths (MEPs) were computed for each reaction. The MEPs give a complete description of the process, in terms of both mechanism and rate limiting energy barrier, and yield a molecular movie of each hydrolysis reaction (available in Additional file 1).

Our results show that the mechanism of hydrolysis is very dependent on the location of the protons on the triphosphate: Protonation of the α- and β-phosphate groups favors the sequential mechanism, whereas protonation on the γ-phosphate favors a concurrent reaction. This can be explained in terms of the charge shifts that occur during these different reaction mechanisms. Overall, the lowest barriers are achieved with the sequential mechanism, which can be explained by the fact that the dissociated P_γ_O_3_^−^ metaphosphate intermediate (Fig. 2d) is a better target for the subsequent attack by the lytic water W_a_ than the –P_γ_O_4_^2−^ group in the concurrent reaction (Fig. 1). This is consistent with the catalytic mechanism in NTPases, which has been shown to proceed via a sequential reaction [7, 21–26]. The present results help to explain why the many H-bonds made by the P-loop to the α- and β-phosphates of the NTP favor such a sequential mechanism in NTPases.

## Results

### Reaction paths

**Fig. 3:**
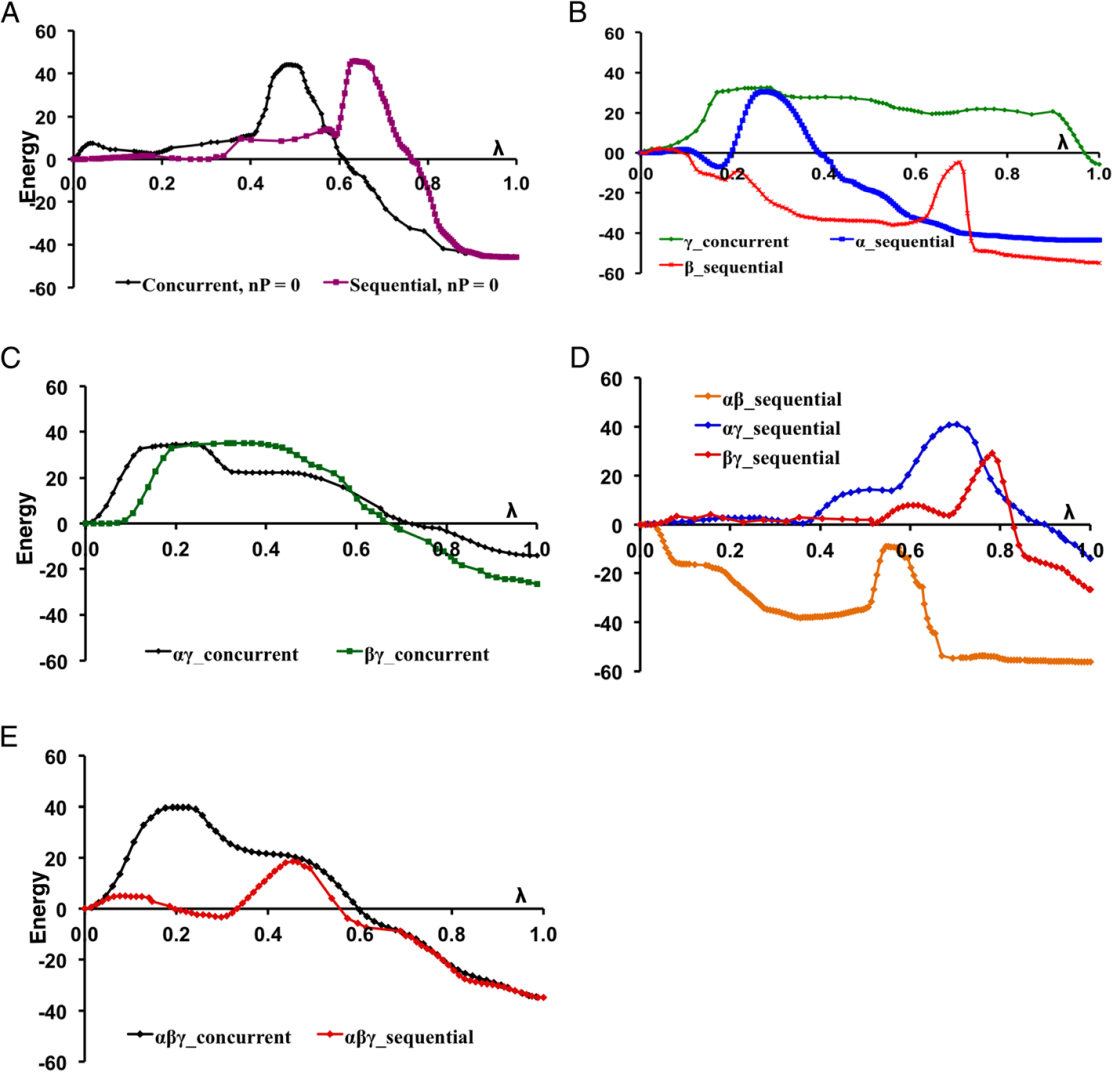
Energy profiles (in kcal mol^−1^) for methyl triphosphate hydrolysis in different protonation states. **a** No protons on the triphosphate (n_P_ = 0). **b** Single protonation (n_P_ = 1). Double protonation (n_P_ = 2) resulting in **c** concurrent reactions or **d** sequential reactions. **e** triple protonation (n_P_ = 3). Greek letters (α,β,γ) indicate on which phosphate group the proton is located (see Fig. 1 for nomenclature). The energy barriers seen here correspond to those listed in Tables 1 and 2. The energy is plotted as a function of the curvilinear reaction coordinate (λ), which measure the progress of the reaction as the sum of conformational changes that occur along the MEP (in terms of RMS-change in all atomic coordinates), starting from the reactant state (λ = 0). Here λ is normalized by the total length of the curvilinear MEP, so that the hydrolysis product has λ = 1

To obtain one concurrent and one sequential reaction path for each of the eight protonation states mentioned above, initial constraints on the atomic coordinates were applied to channel the refinement of the minimum energy paths (MEP) into a corresponding valley of the potential energy surface. However, after all constraints are removed, it turns out that there are not 16 such MEPs. Only 12 MEPs could be found on the respective potential energy landscapes: 5 concurrent MEP (whose energy barriers are listed in Table 1) and 7 sequential MEPs (listed in Table 2). For the concurrent reaction, there is no MEP on the energy landscape for protonation states α, β, and αβ, while for the sequential reaction, no MEP could be found for single protonation on γ. The explanation for this is given below. The energy profiles along the 12 MEPs are shown in Fig. 3. In the following sections, the characteristics of these paths are presented, starting with the concurrent paths and continuing with the sequential paths. Twelve molecular movies showing the conformational changes that occur during these MEPs are available as Supplemental Information.

**Table 1:** Effect of protonation on the barrier ΔEǂ of the concurrent reaction ^[a]^

n_P_ = 0	n_P_ = 1		n_P_ = 2		n_P_ = 3	
44.0	α:	^[b]^	αγ:	34.5	αβγ:	39.9
	β:	^[b]^	αβ:	^[b]^		
	γ:	32.4	βγ:	35.1		

^[a]^ ΔE^ǂ^ is the energy (AM1/d in kcal mol^−1^) difference between the optimized reactant and the rate limiting transition state (saddle point). n_P_ is the number of protons on the triphosphate (α, β and γ indicate which phosphate group is protonated). ^[b]^ Various searches for a concurrent reaction failed (hydrolysis always proceeded through a sequential mechanism, see Table 2)

**Table 2:** Effect of protonation on the barriers of the sequential reaction ^[a]^

n_P_^[b]^		Δ*E*_1_^ǂ [c]^	*E*_meta [d]_	*E*_2 [e]_	Δ*E*_2_^ǂ [f]^
0		14.2	11.2	45.9	34.7
1	α:	1.82	−7.07	30.4	37.5
1	β:	1.99	−36.0	−4.6	31.4
1	γ: ^[g]^	---	---	---	---
2	αβ:	0.34	−38.2	−9.0	29.2
2	αγ:	14.5	13.9	40.9	27.0
2	βγ:	8.02	3.8	29.1	25.3
3	αβγ:	4.9	−3.27	18.6	21.9

^[a]^ Energy barriers (AM1/d, in kcal mol^−1^) along the sequential reaction for different protonation states. ^[b]^ n_P_ is the number of protons on the triphosphate (α, β and γ indicate which phosphate is protonated). ^[c]^ Δ*E*_1_^ǂ^ is the barrier of the metaphosphate formation, i.e., the energy difference between the saddle point for P_γ_-O_βγ_ bond cleavage and the reactant. Note that Δ*E*_1_^ǂ^ is the same as *E*_1_ defined in the main text. ^[d]^ *E*_*meta*_ is the energy of the geometry-optimised metaphosphate intermediate, relative to the initial reactant. ^[e]^ *E*_2_ is the energy of the saddle point for W_a_ attack onto the metaphosphate, relative to the reactant. ^[f]^ Δ*E*_2_^ǂ^ = (*E*_2_ - *E*_meta_) is the activation barrier for water W_a_ attack onto the metaphosphate. ^[g]^ Various searches for a sequential reaction failed (hydrolysis always proceeded through a concurrent mechanism)

### Concurrent paths

#### Unprotonated case (n_p_ = 0)

**Fig. 4:**
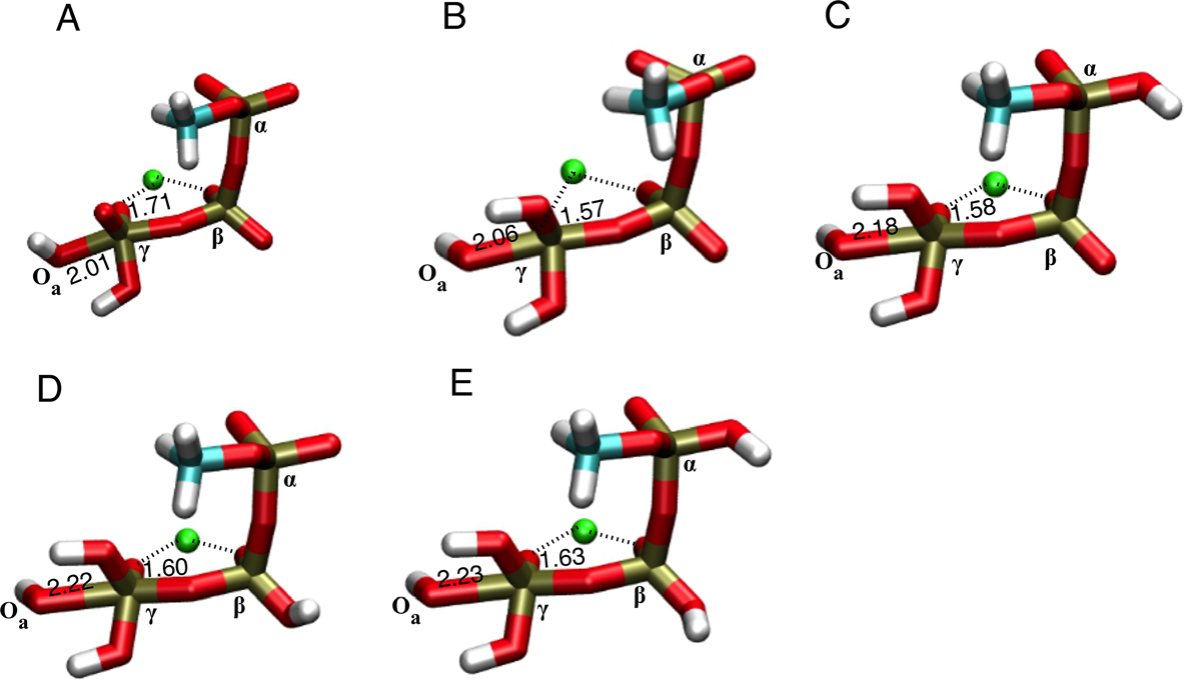
Transition state in the concurrent reactions. **a** Methyl triphosphate fully deprotonated (number of protons n_P_ = 0), **b** γ-protonated (n_P_ = 1), **c** αγ-protonated (n_P_ = 2), **d** βγ-protonated (n_P_ = 2) and **e** αβγ-protonated (n_P_ = 3). See also caption of Fig. 3. Coordination bonds to Mg^2+^ are shown as *dotted line*. The four water molecules coordinating the Mg^2+^ are not shown, but are present in the calculations. The distance from the P_γ_ to the leaving and attacking oxygen atoms is indicated in Å

The energy profile of the concurrent MEP obtained in absence of any protons on the triphosphate is plotted in Fig. 3a. It has a rate-limiting barrier of 44 kcal mol^−1^ about half-way (λ = 48 %, see legend of Fig. 3 for a definition of λ) along the MEP. The structure of that transition state is shown in Fig. 4a. The γ-phosphorus has a distance to the leaving oxygen O_βγ_ (see Fig. 2c for atomic nomenclature) of 1.71 Å and a distance to the oxygen of the attacking water W_a_ equaling 2.01 Å. These two short distances reflect the fact that the breaking of the P_γ_–O_βγ_ bond is concerted with the formation of the O_a_–P_γ_ bond characteristic for a concurrent mechanism. In molecular movie C1 (Additional file 2), it can be seen that shortly before the transition state is reached, one of the two protons of water W_a_ is transferred onto one of the oxygens of the γ-phosphate, which acts here as a “catalytic base”. The high-energy barrier is due to the fact that two strong bonds are being broken nearly simultaneously (Fig. 1): the P_γ_–O_βγ_ bond and the O_a_–H bond of water W_a_. Moreover, the γ-phosphate group (−OP_γ_O_3_^2−^) formally bears two negative charges, which makes it a poor target for the nucleophilic attack by the O_a_–H^−^ moiety of water W_a_. Also, the tetrahedral geometry of the –OP_γ_O_3_^2−^ group does not allow for a close approach of the phosphorus by water W_a_.

#### Single protonation (n_P_ = 1)

The energy profile of the concurrent MEP obtained with one proton placed on the γ-phosphate is plotted in Fig. 3b. The rate limiting energy barrier is 32.4 kcal mol^−1^ (located 27 % along the MEP) and its structure is shown in Fig. 4b. In spite of the fact that this structure and the reaction (see Additional file 3: Movie C2) are very similar to the un-protonated concurrent case (compare Fig. 4a and b), adding a proton on the γ-phosphate significantly lowers (by 11.6 kcal mol^−1^, Table 1) the activation barrier of concurrent hydrolysis. This can be explained by the fact that the protonated γ-phosphate (−OP_γ_O_3_H^−^) bears one less negative charge than the un-protonated –OP_γ_O_3_^2−^, so that its repulsion with the partial negative charge on oxygen O_a_ of water W_a_ is diminished. In the cases of single protonation on either the α- or β-phosphates, it was not possible to isolate concurrent MEPs. In spite of using initial constraints, the MEPs always reverted to a sequential reaction (presented below).

#### Double protonation (n_P_ = 2)

The energy profiles of the concurrent reaction obtained with two protons placed on the αγ- or βγ-phosphates are plotted in Fig. 3c. Structurally, these reactions proceed (Additional file 4: Movie C3 and Additional file 5: Movie C4) very similarly to the un-protonated and the γ-protonated concurrent cases. Their transition state structures (Fig. 4c and d, respectively) are very similar to the other concurrent transition states (Fig. 4). Their energy barriers (34.5 kcal mol^−1^ for αγ and 35.1 kcal mol^−1^ for βγ) are slightly higher (2–3 kcal mol^-1^, Table 1) than the barrier of the singly protonated case. This may be explained by the fact that α- or β-protonation favors a dissociation of the γ-phosphate (as will be shown below), which involves larger P_γ_-O_βγ_ distances than those of the concurrent transition state for single γ-protonation. Indeed, the P_γ_–O_βγ_ distance is 1.58 Å and 1.60 Å for the αγ and βγ protonation, respectively, slightly larger than the 1.57 Å for single γ-protonation (Fig. 4). Thus, adding α- or β-protons on the concurrent transition states raises the barrier relative to the singly γ-protonated triphosphate. For the same reason, double protonation on the αβ phosphates always resulted in a sequential MEP, in spite of attempts to use initial constraints towards a concurrent reaction.

#### Triple protonation (n_P_ = 3)

The energy profile of the concurrent MEP with triply (α,β,γ) protonated triphosphate is plotted in Fig. 3e. The transition state (Fig. 4e) has an even larger P_γ_–O_βγ_ distance (1.63 Å) than the αγ or βγ doubly protonated transition states (Fig. 4c and d). The unfavorable effect of the α and β protons on the concurrent energy barrier (described above) is more than cumulative, since it is 7.5 kcal mol^-1^ higher than for single γ-protonation (Table 1). Additional file 6: Movie C5 shows the structural changes during this reaction.

### Sequential paths

**Fig. 5:**
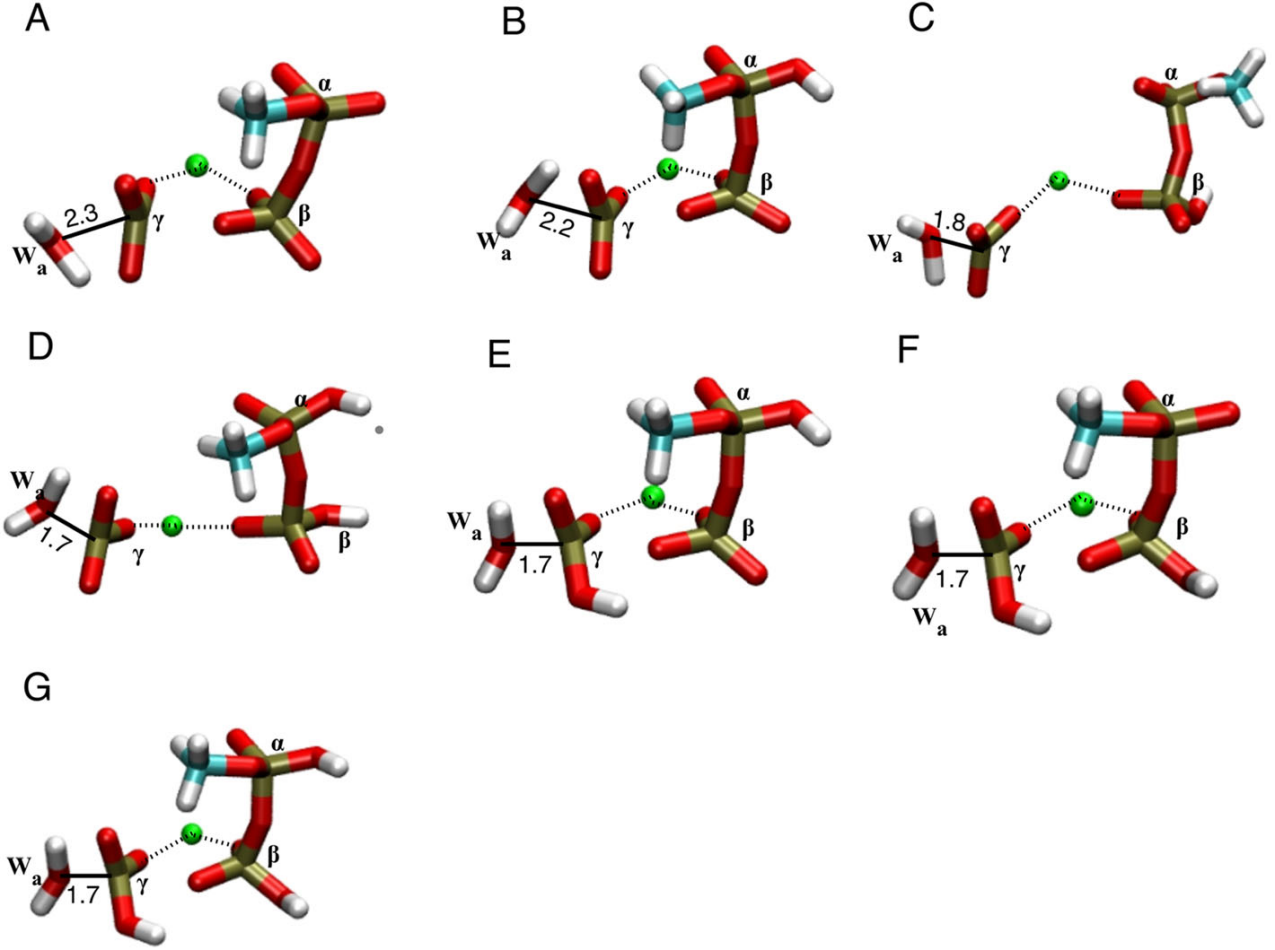
Transition state of the second step (W_a_ attack onto γ-metaphosphate) in sequential reactions. **a** Methyl triphosphate fully de-protonated (number of protons n_P_ = 0), **b** α-protonated (n_P_ = 1), **c** β-protonated (n_P_ = 1), **d** αβ-protonated (n_P_ = 2), **e** αγ-protonated (n_P_ = 2), **f** βγ-protonated (n_P_ = 2) and **g** αβγ-protonated (n_P_ = 3). The distance between the oxygen atom of water W_a_ and the γ-phosphorus (*solid line*) is given in Å. The coordination bonds to the Mg^2+^ are shown as *dotted line*. The four water molecules coordinating the Mg^2+^ are not shown, but are present in the calculations

All concurrent MEPs described above have one high rate-limiting transition state (shown in Fig. 4), which belongs to the simultaneous breaking of the P_γ_-O_βγ_ bond and attack of water W_a_ (Fig. 1). In contrast, sequential MEPs have two distinct transition states: The first one belongs to the breaking of the P_γ_-O_βγ_ bond to form a planar metaphosphate (for example, P_γ_O_3_^−^ for n_P_ = 0) intermediate (Fig. 2c), followed by the second transition state which pertains to the breaking of water W_a_ (Fig. 2d) and attack onto the P_γ_O_3_^−^ molecule (for example, to form the H_2_P_γ_O_4_^−^ for n_P_ = 0). The two corresponding saddle points were geometry-optimized for each sequential MEP. Their energies (relative to the reactant state) are called here E_1_ and E_2_, respectively for the first and second transition state (listed in Table 2). For the first transition state, the distances of the γ-phosphorus to the leaving and attacking oxygens are listed in Table 3. The structure of the 2nd transition state of each sequential MEP is shown in Fig. 5. The metaphosphate intermediate state was also geometry optimized and its energy is called here *E*_meta_ (so that the barrier of the second step is Δ*E*_2_^ǂ^ = *E*_2_ - *E*_meta_).

**Table 3:** Inter-atomic distances [Å] in the saddle point of metaphosphate formation ^[a]^

n_P_^[b]^		P_γ_-O_βγ_^[c]^	P_γ_-O_a_^[d]^
0		2.32	2.83
1	α	1.92	2.47
1	β	1.72	2.51
2	αβ	1.85	2.45
2	βγ	2.02	2.34
2	αγ	2.12	2.33
3	αβγ	1.98	2.40

^[a]^ Structures corresponding to the energy Δ*E*_*1*_^ǂ^ in Table 2. ^[b]^ n_P_ is the number of protons on the triphosphate (α, β and γ indicate which phosphate is protonated. ^[c]^ Distance between P_γ_ and the leaving oxygen O_βγ_ (see Fig. 1d for atomic nomenclature). ^[d]^ Distance between P_γ_ and the attacking oxygen of water W_a_

#### Unprotonated case (n_P_ = 0)

The energy profile of the sequential MEP obtained without protons on the triphosphate is plotted in Fig. 3a. Barrier Δ*E*_1_^ǂ^ is 14.1 kcal mol^−1^ at λ = 57 %. The energy of the metaphosphate intermediate is *E*_meta_ = 11.2 kcal mol^−1^, so that the second barrier (at λ = 64 %) is Δ*E*_2_^ǂ^ = 45.9-11.2 = 34.7 kcal mol^−1^ (Table 2). Thus, step 1 (the breaking of the P_γ_-O_βγ_ bond) has a much lower barrier than step 2 (water attack). This turns out to be the case for all other sequential MEPs (see Table 2). The transition state for step 2 is shown in Fig. 5a, in which the planar P_γ_O_3_^−^ molecule is clearly recognizable and the O_a_–P_γ_ distance is short at 2.3 Å. The sequentiality of step 1 and step 2 can be seen in Additional file 7: Movie S1. Relative to the reactant, the energy (*E*_2_ = 45.9 kcal mol^−1^) of the rate-limiting transition state is similar to that of the un-protonated concurrent reaction (Δ*E* ^ǂ^ = 44.0 kcal mol^−1^, Table 1). Thus, in absence of protons, there is no clear preference for either the concurrent or the sequential mechanism of hydrolysis.

#### Single protonation (n_P_ = 1)

The energy profiles of the sequential MEP obtained with one proton placed on either the α- or the β-phosphate are plotted in Fig. 3b. In both cases, the effect of these protonations on the barrier of the first step (P_γ_-O_βγ_ bond breaking) is very strong, lowering Δ*E*_1_^ǂ^ by ~12 kcal mol^−1^ relative to the un-protonated case: Δ*E*_1_^ǂ^ = 1.82 kcal mol^−1^ (at λ = 9 %) for α- and Δ*E*_1_^ǂ^ = 1.99 kcal mol^−1^ (at λ = 4 %) for β-protonation (Table 2). Such low barriers mean that the dissociation of the P_γ_–O_βγ_ bond is nearly unhindered at room temperature when the triphosphate is protonated on the α- or β-positions, a remarkable result. Moreover, the resulting metaphosphate intermediates are more stable than the reactant state (*E*_meta_ < 0, Table 2).

How can protonation of the αβ-diphosphate moiety promote metaphosphate dissociation in such a dramatic way? The answer lies in the change of charge distribution upon dissociation: For example, in the un-protonated triphosphate reactant, two formal negative charges are located on the γ-phosphate (-OP_γ_O_3_^2−^) and two negative charges are on the αβ-diphosphate moiety (Fig. 2c). In the metaphosphate intermediate, the P_γ_O_3_^−^ bears a single negative charge and the αβ-diphosphate has three negative charges (Fig. 2d). Thus, one negative charge shifts from the γ- to the αβ-phosphates upon dissociation. This charge shift is strongly favored when a positive charge (H^+^) is placed onto the αβ-moiety.

The barrier of step 2 (breaking of water W_a_) is Δ*E*_2_^ǂ^ = 37.5 kcal mol^−1^ for α- and Δ*E*_2_^ǂ^ = 31.4 kcal mol^−1^ for β-protonation (Table 2), which is similar to that of the un-protonated case (Δ*E*_2_^ǂ^ = 34.7 kcal mol^-1^). This indicates that the effect of these protonations on step 2 is limited, as would be expected from the fact that the αβ-moiety is no longer covalently bound to the γ-phosphate when the later gets attacked by water W_a_ (see the corresponding transition states in Fig. 5b and c). Note that it is difficult to compare the energy of the metaphosphate intermediate (*E*_meta_) of the α- and β-cases, due to the large conformational change that occurs (from λ = 21 to 58 %) after metaphosphate formation in the β-case (compare Additional file 8: Movie S2 and Additional file 9: Movie S3).

All attempts to obtain a sequential MEP with one proton placed on the γ-phosphate failed, because the reaction always became concurrent. This effect can be explained in terms of the above mentioned charge shift: Placing a positive charge (H^+^) on the γ-phosphate strongly disfavors the shift of the negative charge from the γ- to the αβ-moiety (Fig. 2c → d) that needs to take place upon metaphosphate dissociation. Therefore, placing a proton on the γ-phosphate destabilizes the metaphosphate so much, that the “transition state” of step 1 is no longer a saddle-point on the energy surface (consequently, a MEP can not be found).

#### Double protonation (n_P_ = 2)

Placing two protons on the αβ-moiety (one on the α-, the other on the β-phosphate) favors the shift of negative charge from the γ- to αβ-phosphates even more than a single proton. Not surprisingly, the barrier of metaphosphate dissociation drops even lower in this case, Δ*E*_1_^ǂ^ = 0.34 kcal mol^−1^ (Table 2). The corresponding energy profile is plotted in Fig. 3d, where this barrier (located at λ = 2 %) can be seen to be so low that the first step is essentially barrier-less. After some rearrangements of the metaphosphate intermediate (from λ = 2 to 35 %, see also Additional file 10: Movie S4), the barrier of step 2 (breaking of water W_a_) is Δ*E*_2_^ǂ^ = 29.2 kcal mol^−1^ (transition state shown in Fig. 5d), which is similar to the value obtained for single protonation on β (Δ*E*_2_^ǂ^ = 31.4 kcal mol^−1^, Table 2) and confirms that protonation of the αβ-moiety does not significantly affect step 2 of the sequential reactions.

Double protonation on the αγ- or βγ-phosphates has a very different effect than the double protonation of the αβ-moiety described above, as can be seen by comparing their three energy profiles (Fig. 3d). Indeed, the resulting barrier for step 1 is Δ*E*_1_^ǂ^ = 14.5 kcal mol^−1^ for αγ-protonation, as high as for step 1 in the un-protonated case (Δ*E*_1_^ǂ^ = 14.2 kcal mol^−1^ Table 2), and much higher than for the singly α-protonated case (Δ*E*_1_^ǂ^ = 1.82 kcal mol^−1^). Thus, protonation on the γ-phosphate counteracts the promoting effect of α-protonation on the metaphosphate dissociation step. As described above, this is due to the unfavorable effect of the positive charge (H^+^) on the γ-position, which opposes the charge shift from the γ- to the αβ-moieties, thereby suppressing the favorable effect of α-protonation. The same behavior happens for double protonation on βγ, for which Δ*E*_1_^ǂ^ = 8.02 kcal mol^−1^ (Table 2). This is not as high as for αγ-protonation, but still significantly higher than the single β-protonation (Δ*E*_1_^ǂ^ = 2.0 kcal mol^-1^, Table 2). The fact that βγ- has a smaller Δ*E*_1_^ǂ^ than αγ-protonation shows that the promoting effect on dissociation is stronger for protonation on the β-phosphate than on the α-phosphate. This is not surprising, since the formal charge change upon dissociation is larger on the β-phosphate (1e → 2e) than on the α-phosphate (1e → 1e), as illustrated by the charge distributions of Fig. 2c and d.

The Δ*E*_2_^ǂ^ barrier of step 2 (W_a_ water attack) is lower for the αγ- and βγ-cases, 27.0 and 25.3 kcal mol^-1^ respectively (for structures shown in Fig. 5e and f), than for single protonation on α or β (37.5 and 31.4 kcal mol^−1^ respectively, see Table 2). The reason for this is the same as the one given above to explain barrier lowering in the concurrent singly γ-protonated case: The presence of a γ-proton leads to a dissociated metaphosphate in the neutral HP_γ_O_3_ form (instead of the negatively charged P_γ_O_3_^-^ form). This generates less repulsive interactions with the partial negative charge on oxygen O_a_ of water W_a_ when it attacks this neutral metaphosphate in step 2. Thus, just as seen in the concurrent reactions, having a proton on the γ-phosphate facilitates the activation of water W_a_ when it attacks the γ-phosphorus. Additional file 11: Movie S5 and Additional file 12: Movie S6 show the sequential αγ- and βγ-reactions, respectively.

#### Triple protonation (n_P_ = 3)

The energy profile of the sequential MEP with a triply (α,β,γ) protonated triphosphate is plotted in Fig. 3e. The first barrier Δ*E*_1_^ǂ^ = 4.9 kcal mol^−1^ (Table 2) is the result of a compromise between the favorable (i.e. decreasing Δ*E*_1_^ǂ^) effect of having two protons on the αβ-moiety (which favors the charge shift in Fig. 2c → d), and the unfavorable (i.e. raising Δ*E*_1_^ǂ^) effect of having a proton on the γ-phosphate (which disfavors the charge shift).

The transition state of step 2 (Fig. 5g) gives a barrier Δ*E*_2_^ǂ^ = 21.9 kcal mol^−1^. This is the lowest of all Δ*E*_2_^ǂ^ values (Table 2), which can be related to the fact that an increase in the total number of protons (i.e., a decrease in the net negative charge) on the triphosphate leads to less repulsion with the O_a_H^−^ group that attacks the γ-phosphorus in step 2. Indeed, the values of Δ*E*_2_^ǂ^ gradually drop as one looks down the right column of Table 2. Overall, the triply protonated sequential MEP has the lowest rate limiting barrier of all paths examined here, and is shown in Additional file 13: Movie S7.

## Discussion

### Principle effects of (α, β or γ)-protonation

The results obtained here for the different protonations of triphosphate can be explained by the four following principles:

1. α- and/or β-protonation favor the sequential reaction
  When a single or two protons are placed on the α- and/or β-positions, hydrolysis is found to occur only via the sequential mechanism. Concurrent transition states can’t be located for protonation cases α, β or αβ. The energy barrier for P_γ_–O_βγ_ bond breaking, Δ*E*_1_^ǂ^, is considerably lower for protonation cases α, β (Δ*E*_1_^ǂ^ ~ 2 kcal mol^−1^) and αβ (Δ*E*_1_^ǂ^ ~ 0.34 kcal mol^−1^) than for the dissociation barrier of the un-protonated case (Δ*E*_1_^ǂ^ = 14.2 kcal mol^−1^), see Fig. 6a. All this shows that P_γ_–O_βγ_ bond dissociation is strongly favored when protons are added onto α or β positions. This is because α- and/or β-protonation favors the negative charge shift from the γ-phosphate to the α,β-diphosphate upon P_γ_–O_βγ_ bond breaking (Fig. 2c → d): In un-protonated triphosphate, two formal negative charges reside on the αβ-diphosphate and two negative charges on the γ-phosphate. After P_γ_–O_βγ_ bond cleavage, the αβ-diphosphate moiety bears three negative charges and the γ-metaphosphate has one negative charge. The positive charge of a proton placed either on an α or a β oxygen atom of triphosphate pulls the electron density away from the γ-phosphate group and towards the αβ-diphosphate moiety. This prepares for the electronic distribution of the dissociated metaphosphate state, thus explains the large reduction in the Δ*E*_1_^ǂ^ energy barrier. This stabilization effect is so strong that the resulting metaphosphate state constitutes a stable intermediate in the energy profile of all sequential reactions (see Fig. 3). The energy *E*_meta_ of this intermediate is lower than the reactant (*E*_meta_ < 0) for all cases of protonation on exclusively α- and/or β-groups (Table 2). As is expected from the formal charge distribution of the dissociated state (Fig. 2d), this pulling effect on the electron-density towards the αβ-diphosphate is somewhat stronger for β-protonation than for α-protonation (lower values of *E*_meta_ for β-protonation in Table 2).

**Fig. 6:**
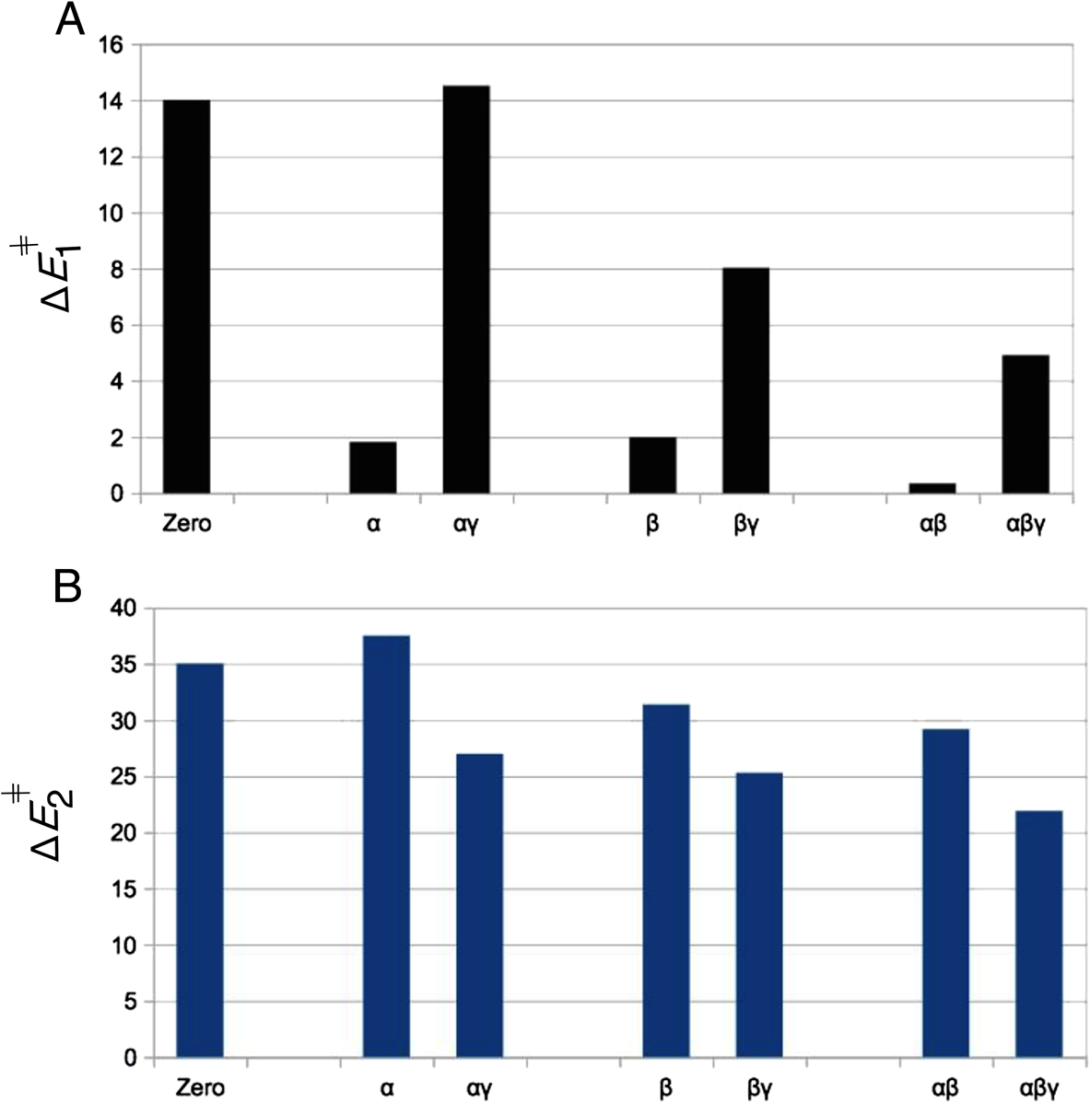
Effect of protonation on the two steps of the sequential mechanism. Energy barriers **a** Δ*E*_1_^ǂ^ for breaking the P_γ_-O_βγ_ bond and **b** Δ*E*_2_^ǂ^ for breaking of water W_a_ (as given in Table 2). Greek letters (α,β,γ) indicate on which phosphate the proton is located (for example αγ means αγ-protonation). Zero means that there are no protons on the triphosphate (n_P_ = 0)
2. γ-protonation disfavors the sequential reaction
  The effect of γ-protonation is the opposite of the effect of α/β-protonation and raises the Δ*E*_1_^ǂ^ energy barrier of P_γ_–O_βγ_ bond cleavage. When a γ-proton is added to an α-protonated triphosphate, Δ*E*_1_^ǂ^ raises from 1.82 kcal mol^−1^ to 14.5 kcal mol^−1^ (Fig. 6a). A similar trend is observed when the γ-proton is added onto the β-protonated triphosphate (with Δ*E*_1_^ǂ^ raising from 1.99 kcal mol^−1^ to 8.02 kcal mol^−1^), or when added to the αβ-protonated triphosphate (Δ*E*_1_^ǂ^ increases from 0.34 kcal mol^−1^ to 4.9 kcal mol^−1^), see Fig. 6a. Again, this disfavoring effect of γ-protonation on P_γ_–O_βγ_ bond breaking is explained by the charge-shift during P_γ_–O_βγ_ bond cleavage. The positive charge of the γ-proton hinders the shift of the negative charge from the γ-phosphate to the αβ-diphosphate (Fig. 2c → d), thus disfavoring dissociation and raising Δ*E*_1_^ǂ^. This effect is strongest in absence of any protons on α and β. In that case, a single proton on γ-position even abolishes the sequential reaction (as mentioned in Results, a stable transition state can not be found).
  Conversely, γ-protonation is necessary to be able to observe a concurrent reaction at all. Indeed, all concurrent paths with protonated triphosphate have a proton on the γ-phosphate (Table 1). In absence of γ-proton, these MEPs all revert to the sequential mechanism. For example, there is no concurrent MEP for singly α-protonated triphosphate, but a concurrent reaction can be found for the αγ-protonated case (Table 1). The same effect is observed for adding a γ-proton to β-protonated triphosphate, or for adding a γ-proton to the αβ-protonated triphosphate. Thus, adding a γ-proton alters the potential energy surface in such a way that the concurrent mechanism becomes feasible.
3. γ-protonation favors the breakup of water W_a_
  For the sequential reactions, γ-protonation lowers the activation barrier Δ*E*_2_^ǂ^ of water attack: For example, the Δ*E*_2_^ǂ^ of α-protonated triphosphate decreases from 37.5 to 27.0 kcal mol^−1^ in αγ-protonated triphosphate, a reduction of 10.5 kcal mol^−1^ upon addition of the γ-proton (see Fig. 6b). Similarly, Δ*E*_2_^ǂ^ of β-protonated methyl triphosphate (31.4 kcal mol^−1^) reduces to 25.3 kcal mol^−1^ upon addition of the γ-proton, and addition of the γ-proton to αβ-protonated triphosphate reduces the barrier from 29.2 to 21.9 kcal mol^−1^ (Fig. 6b). The reason for this effect is that γ-protonation yields a neutral metaphosphate intermediate of the form HP_γ_O_3_. It is not negatively charged (unlike the un-protonated P_γ_O_3_^−^), which reduces the electrostatic repulsion between the partial negative charge on oxygen O_a_ of water W_a_ and the γ-metaphosphate. Therefore, the γ-protonated metaphosphate is a better target for nucleophilic attack by W_a_ (and its accompanying breakup) than the un-protonated PO_3_^−^. The same effect is at work in the concurrent reactions, where all reactions for triphosphate with a γ-proton can be seen (in Table 1) to have a lower activation barrier Δ*E* ^ǂ^ than the un-protonated (n_P_ = 0) case (Δ*E* ^ǂ^ = 44 kcal mol^−1^). Thus, for both concurrent and sequential mechanisms, protonation in the γ-position lowers the energy barrier of water breakup and attack.
4. The sequential mechanism yields lower rate-limiting barriers
  Even though Δ*E*_2_^ǂ^ (the barrier of the second step in sequential pathways) is always higher than the Δ*E*_1_^ǂ^ barrier (i.e., Δ*E*_2_^ǂ^ > Δ*E*_1_^ǂ^, see Table 2), Δ*E*_2_^ǂ^ is always lower than the rate-limiting barrier Δ*E* ^ǂ^ of the corresponding (i.e., for a given protonation state) concurrent pathway (compare Tables 1 and 2). For example for αγ-protonation, the sequential Δ*E*_2_^ǂ^ (=27.0 kcal mol^−1^) is 7.5 kcal mol^−1^ lower than the concurrent Δ*E* ^ǂ^ (=34.5 kcal mol^−1^). Likewise for the un-protonated case (n_P_ = 0), where Δ*E*_2_^ǂ^ (=35 kcal mol^-1^) is 9 kcal mol^−1^ less than Δ*E* ^ǂ^ (=44 kcal mol^−1^). This Δ*E*_2_^ǂ^ < Δ*E* ^ǂ^ rule applies also in the other protonation cases, βγ and αβγ. One explanation is that the metaphosphate molecule P_γ_O_3_^−^ (or HP_γ_O_3_ if γ–phosphate was protonated) generated by dissociation step 1 (Fig. 2c) is a much better target for water W_a_ than the –OP_γ_O_3_^2-^ (or –OP_γ_O_3_H^-^) group of un-dissociated triphosphate, for two reasons: a) The metaphosphate is planar, a geometry that allows closer approach of the attacking water W_a_ than the tetrahedral –O–PO_3_ group. b) The metaphosphate has one less negative charge than the un-dissociated –OPO_3_ group (for a given protonation of the γ-phosphate), thus generating less electrostatic repulsion with the negative partial charge on oxygen O_a_ of water W_a_. The other reason for having Δ*E*_2_^ǂ^ < Δ*E* ^ǂ^ is that in the concurrent mechanism, the energetic cost of the breaking water W_a_ and breaking the P_γ_O_βγ_ bond are paid simultaneously in a single transition state, while in the sequential mechanism, these costs are spread over two transition states.

### Implications for triphosphate hydrolysis in enzymes

#### Catalytic strategy

There are clear parallels between the hydrolytic reaction in enzymes and the αβγ-protonation case described above in terms of the resulting sequential mechanism and the respective energy barriers: The lowest barrier of hydrolysis in vacuum is obtained here when all three phosphates (α,β,γ) of triphosphate are protonated, with a sequential mechanism (Δ*E*_1_^ǂ^ = 4.9 kcal mol^−1^ and Δ*E*_2_^ǂ^ = 21.9 kcal mol^−1^). Likewise, it has been shown recently for several NTPases (myosin, [7, 21] kinesin, [24] F_1_-ATPase, [22] RAS-GAP [27, 28] ) that they all have a catalytic mechanism that is sequential, involving the initial formation of a P_γ_O_3_^−^ metaphosphate, followed by the attack of the lytic water (which is always placed like W_a_ in Fig. 2a and b). This similarity is not due to protonation of the phosphates in the enzyme (computational studies indicate that the triphosphate is fully deprotonated when bound in the catalytic pocket), [21] but arises because interaction of each phosphate group with its positively charged protein environment promotes similar charge-shifts within the triphosphate as those described above for the protonation in vacuum. For example in myosin, the six hydrogen bonds of the P-loop are all made to the αβ-phosphates (see Fig. 2a), thus pulling negative charge away from the P_β_-O_βγ_ bond. This is likely to have a similar lowering effect on the Δ*E*_1_^ǂ^ barrier as the charge shift induced by the protonation on the α and/or β phosphates (which lowers Δ*E*_1_^ǂ^ by 12–14 kcal mol^−1^, see Fig. 6a). The three H-bonds donated to the γ-phosphate by the Ser181 side chain and the backbone of Ser236 and Gly457 (Fig. 2a) make the γ-phosphorus become a better target for nucleophilic attack by water W_a_, probably contributing to the lowering of Δ*E*_2_^ǂ^ in the enzyme in a similar way as protonation of the γ-phosphates (which lowers Δ*E*_2_^ǂ^ by 6–10 kcal mol^-1^, see Fig. 6b). A crude estimate of the amount of positive charge placed in direct contact with the triphosphate in myosin can be made: the nine H-bonds and Lys185^+^ amount to approximately +3.25 charges (counting ~0.25 charge per H-bond, which is the typical partial atomic charge on a proton of backbone NH groups in classical force-fields) [29]. Of course this number is not a true net charge (it neglects the counter-charge of each H-bond dipole), but it is close to the +3 charge of three protons distributed onto the α-, β- and γ-protonated triphosphate. Thus it is not surprising that the Δ*E*_1_^ǂ^ barrier obtained for myosin (8.7 kcal mol^−1^) is nearly as low as the Δ*E*_1_^ǂ^ obtained with triply protonated triphosphate in vacuum (4.9 kcal mol^−1^).

In the present vacuum simulations, the proton abstracted from W_a_ was transferred to the γ-phosphate (in both the concurrent and sequential reactions). Thus the γ-phosphate serves as both the proton acceptor and as the general base that activates water W_a_. In the enzymes, the final acceptor of the proton is also the dissociated γ-phosphate, but the NTPases utilize an external catalytic base (e.g., Glu459 in myosin, see Fig. 2a) to activate water W_a_. This external base serves to polarize water W_a_, either directly (in RAS-GAP [27]) or via an intercalating helping water molecule (W_h_ in Fig. 2a). This allows the enzymes to further lower the barrier for lysis of water W_a_, and thus facilitate water attack onto the dissociated metaphosphate. In this way, the barrier for step 2 of the sequential reaction (Δ*E*_2_^ǂ^) can be lowered even more than can be achieved by only having partial positive charges interacting with the γ-phosphate group (described above).

During step 2 of the present sequential pathways in vacuum, the proton abstracted from water W_a_ is directly transferred onto the oxygen of the γ-phosphate. The corresponding transition state contains a 4-membered ring (P_γ_–O_γ_–H_a_–O_a_–P_γ_), which induces some strain. In the enzymes, this transfer occurs via either a helping water (W_h_) and/or the alcohol group of a nearby Serine side chain (for example Ser181 in myosin, see Fig. 2a) [21]. The resulting 6- or 8-membered ring in the transition state allows for less strain, thereby further lowering the barrier Δ*E*_2_^ǂ^. Together with the activation from an external base, this explains how the enzymes manage to bring Δ*E*_2_^ǂ^ down to values as low as 10–17 kcal mol^−1^, [21, 22, 30–32] while in vacuum the lowest value that could be achieved here for Δ*E*_2_^ǂ^ is 21.9 kcal mol^−1^ (for αβγ triple protonation, Table 2).

In all combined quantum mechanical/classical (QM/MM) simulations of ATP hydrolysis in myosin during which a proton had first transferred to the γ-phosphate, the mechanism has always been found to be concurrent, never sequential [5, 33–37]. This is consistent with the results obtained here: Whenever the γ-phosphate is protonated, the P_γ_-O_βγ_ bond is strengthened, preventing a sequential mechanism with prior breaking of the P_γ_-O_βγ_ bond. Conversely, in all those calculations in which the γ-phosphate was not protonated, the mechanism has been observed to be sequential [7, 21, 38]. Similarly, in QM/MM simulations of the phosphoryl transfer reaction in bovine protein tyrosine phosphatase (BPTP), [39] it was shown that the phosphoryl transfer occurs via a sequential mechanism with a 9 kcal mol^−1^ barrier when the leaving phosphate group is unprotonated [39, 40]. In contrast, when the leaving phosphate group was protonated, phosphoryl transfer was seen to occur via a concurrent mechanism with a barrier of 22 kcal mol^−1^ [39]. This can be explained in the same terms as for the effect of γ-protonation on triphosphate hydrolysis: Protonation of the terminal phosphate strengthens the P–O bond, and the P–O bond cleavage become concurrent, thus resulting in a higher barrier.

## Conclusions

A clear set of rules for the effects of α-, β- or γ- protonation on triphosphate hydrolysis in vacuum can be identified. They are: 1) Protonation of the α- or β-phosphate promotes P_γ_–O_βγ_ bond cleavage, thus favoring a sequential reaction. 2) Protonation on γ favors a concurrent reaction, thus disfavoring the sequential pathway. 3) γ-protonation facilitates the attack of water onto the γ-phosphorus. These effects are somewhat additive, so that simultaneous protonation on the γ- and α- (or γ- and β-) positions can result in both concurrent and sequential reactions. These rules can be explained in terms of the charge distribution on the phosphates: i) α- and/or β-protonation pulls electrons towards the α,β-diphosphate moiety, favoring the charge distribution of the dissociated state (Fig. 2d). ii) γ-protonation has the opposite effect, pulling electrons towards the γ-phosphate, favoring the un-dissociated charge distribution (Fig. 2c), thereby making dissociation less favorable. iii) γ-protonation reduces the negative charge of the γ-phosphate, which thus becomes a better target for the nucleophilic attack by water W_a_.

Breaking-up a water molecule is very difficult, and is the rate-limiting step in all pathways. Therefore, the sequential pathways tend to have a lower rate-limiting barrier than the concurrent reactions. One reason is that the energetic cost of breaking the P_γ_–O_βγ_ bond (Δ*E*_1_^ǂ^) has already been paid in the previous step. The other reason is that the dissociated γ-phosphate (Fig. 2d) is planar and less negatively charged than the un-dissociated γ-phosphate (Fig. 2c), making the former a better target for the nucleophilic attack by water W_a_. For this reason, the lowest energy barrier of hydrolysis is obtained for the α,β,γ-protonated case, which combines all effects.

The present calculations are consistent with experimental studies: Uncatalyzed phosphoryl transfer reaction from ATP, GTP and pyrophosphate are suggestive that the beta-gamma-bridging oxygen atom undergoes significant charge-increase (of −0.55 e) [41]. Charge shift was also observed in the RAS catalyzed hydrolysis of GTP in GAP using time-resolved Fourier transform infrared difference spectroscopy [42]. In NTPases, the charge shifts are induced by placing many positive partial charges in direct contact with each of the three phosphate groups. Additionally, the NTPases further lower the rate-limiting barrier of water lysis by placing a residue (such as Glu459 in myosin, Fig. 2a) nearby that acts as a better general base for water activation than the γ-phosphate. The present study helps to better understand the respective role of the many H-bond donors and positive charges in the active site of NTPases. Depending on its placement, each of these groups contributes differently to the catalytic mechanism, according to the set of rules listed above. It is known that ATP-γ-S is far more stable than ATP, [43] and is not easily hydrolyzed by enzymes [44, 45]. An interesting computational study would be to compare the catalysis of ATP-γ-S with that of ATP.

## Methods

In NTPase enzymes, the triphosphate moiety of NTP is complexed with a hexa-coordinated Mg^2+^ cation, therefore Mg^2+^(H_2_O)_4_–coordinated methyl triphosphate (shown in Fig. 1) was used here as substrate. Harmonic distance constraints between the oxygen and hydrogen atoms of magnesium coordinated water molecules were used to prevent undesired proton transfer from these waters to the triphosphate. In all cases studied here, water W_a_ is taken to transfer one of its protons to the nearest phosphate oxygen, namely of the γ-phosphate. This proton transfer mechanism is referred to as direct proton transfer [7, 46]. Reactant and product structures (see Fig. 1) were energy-optimized for each protonation case using the AM1/d semi-empirical method, [47] with phosphorus parameters modified from those of York et. al. [48] and magnesium parameters designed to be combinable with phosphorus, as previously described [20]. The minimum energy paths (MEPs) and all first order saddle point between the reactant and product structures were computed with the Conjugate Peak Refinement (CPR) method, [49] as implemented in the Trek module of CHARMM [29].

CPR finds the MEP by starting from an initial guess of the path, here the linear interpolation between the Cartesian coordinates of reactant and product. The path is treated as a chain of conformers, and this chain is gradually relaxed (by a controlled energy optimization of all the chain conformers) into a valley of the potential energy surface. By applying appropriate external constraints on the distance between the P_γ_ and the attacking (O_a_) and leaving (O_βγ_) oxygen atoms, the potential energy surface was initially shaped to make sure that the desired (either concurrent or a sequential) reaction valley is present for each case of protonation. The CPR search for a MEP was started with this “shaped” potential. Once the path-chain follows the desired valley (for example sequential), the shaping constraints are removed and the path optimization is continued with CPR. This procedure ensures that, when a valley for a given reaction type (for example concurrent) is present on the native (i.e. unshaped) energy surface, then this valley is found. Conversely, if the desired reaction type has no valley on the native potential, then after the shaping constraints are removed, CPR transforms the path-chain until it follows the actual (sequential in this example) reaction valley.

Given the large number of structures that are present along each MEP and that have to be energy-optimized (i.e., not only the transition states were optimized), it was helpful to use a semi-empirical QM method rather than (much slower) DFT methods. Starting from AM1/d parameters developed for phosphate/Mg^2+^ complexes, [20] we further optimized the parameters of phosphorus for the computation of transition states of phosphate hydrolysis (listed in Table S2) [50]. A comparison of the resulting energies for the hydrolysis reaction of dimethyl phosphate (complexed with Mg^2+^ and five water molecules) showed that the AM1/d method can reproduce the relative energy barriers obtained with DFT (B3LYP/6-31++G(d,p)), both for sequential [50] as well as for concurrent [50, 51] mechanisms. Here, we also compared the reactant and the saddle point structures for the concurrent mechanism with fully deprotonated methyl-triphosphate obtained with the B3LYP/6-31 + G** method to those obtained with these optimized AM1/d parameters. They are shown in Additional file 1: Figure S1A and S1B and the corresponding activation barriers are given in Additional file 1: Table S1. The same comparison was done for the sequential mechanism with α-protonated triphosphate (Additional file 1: Figure S1C and S1D, Table S1). The resulting structures and the energy barriers are qualitatively similar, with an error for the activation barrier of less than 10 %, which shows that the present AM1/d method/parameters are adequate to study the effects of protonation on the barriers of triphosphate hydrolysis. Note that we found that the relative energy of the ADP/P_i_ products (i.e., after the crossing of all the barriers, at λ = 1 in Fig. 3) is not reliable with the AM1/d method, which tends to overstabilize the products compared to the reactant energy. It is possible that results of the completely or partly unprotonated triphosphate hydrolysis may alter when treated in solution. This is because these negatively charged triphosphate species would get more stable towards P-O bond cleavage in solutions.

## Abbreviations

ATP, adenosine triphopshate; MEP, minimum energy path; NTP, nucleoside triphsophate

## Additional files

Additional file 1: Supporting Information. **Figure S1.** Comparison of structures optimized with B3LYP/6-31+G** (colored) and AM1/d (gray). **Table S1.** Comparison of the AM1/d and B3LYP/6-31+G** methods. **Table S2.** Modified Phosphorus parameters used in the AM1/d calculations. (DOC 3 mb)
Additional file 2: Movie C1. Concurrent minimum energy path (MEP) for hydrolysis of unprotonated triphosphate (nP = 0). (MPG 1 mb)
Additional file 3: Movie C2. Concurrent MEP for hydrolysis of triphosphate singly protonated (nP = 1) on the γ phosphate. (MPG 595 kb)
Additional file 4: Movie C3. Concurrent MEP for hydrolysis of triphosphate doubly protonated (nP = 2) on α and γ phosphate. (MPG 787 kb)
Additional file 5: Movie C4. Concurrent MEP for hydrolysis of triphosphate doubly protonated (nP = 2) on β and γ phosphate. (MPG 843 kb)
Additional file 6: Movie C5. Concurrent MEP for hydrolysis of triphosphate triply protonated (nP = 3) on α,β and γ phosphate. (MPG 1 mb)
Additional file 7: Movie S1. Sequential MEP for hydrolysis of un-protonated triphosphate (nP = 0). (MPG 876 kb)
Additional file 8: Movie S2. Sequential MEP for hydrolysis of triphosphate singly protonated (nP = 1) on the α phosphate. (MPG 550 kb)
Additional file 9: Movie S3. Sequential MEP for hydrolysis of triphosphate singly protonated (nP = 1) on the β phosphate. (MPG 793 kb)
Additional file 10: Movie S4. Sequential MEP for hydrolysis of triphosphate doubly protonated (nP = 2) on the α,β phosphates. (MPG 793 kb)
Additional file 11: Movie S5. Sequential MEP for hydrolysis of triphosphate doubly protonated (nP = 2) on the α,γ phosphates. (MPG 777 kb)
Additional file 12: Movie S6. Sequential MEP for hydrolysis of triphosphate doubly protonated (nP = 2) on the β,γ phosphates. (MPG 1 mb)
Additional file 13: Movie S7. Sequential MEP for hydrolysis of triphosphate triply protonated (nP = 3) on the α,β,γ phosphates. (MPG 1 mb)
