# Supplementary material for: Effects of protonation on the hydrolysis of triphosphate in vacuum and the implications for catalysis by nucleotide hydrolyzing enzymes
Source: BMC Biochem. 2016 Jun 29;17:12. doi: 10.1186/s12858-016-0068-7 (PMC5157097; doi:10.1186/s12858-016-0068-7)
Supplement: Additional file 1: — Supporting Information. Figure S1. Comparison of structures optimized with B3LYP/6-31+G** (colored) and AM1/d (gray). Table S1. Comparison of the AM1/d and B3LYP/6-31+G** methods. Table S2. Modified Phosphorus parameters used in the AM1/d calculations. (DOC 3 mb) [file 12858_2016_68_MOESM1_ESM.doc]

Supporting Information:

Catalytic effect of protonation on the hydrolysis of triphosphate and its implications for nucleotide hydrolyzing enzymes

Farooq Ahmad Kiani, Stefan Fischer*

AUTHOR ADDRESS. Computational Biochemistry, Heidelberg University, Interdisciplinary Center for Scientific Computing (IWR), Im Neuenheimer Feld-205, D-69120, Heidelberg, Germany.

* correspondance to: stefan.fischer@iwr.uni-heidelberg.de


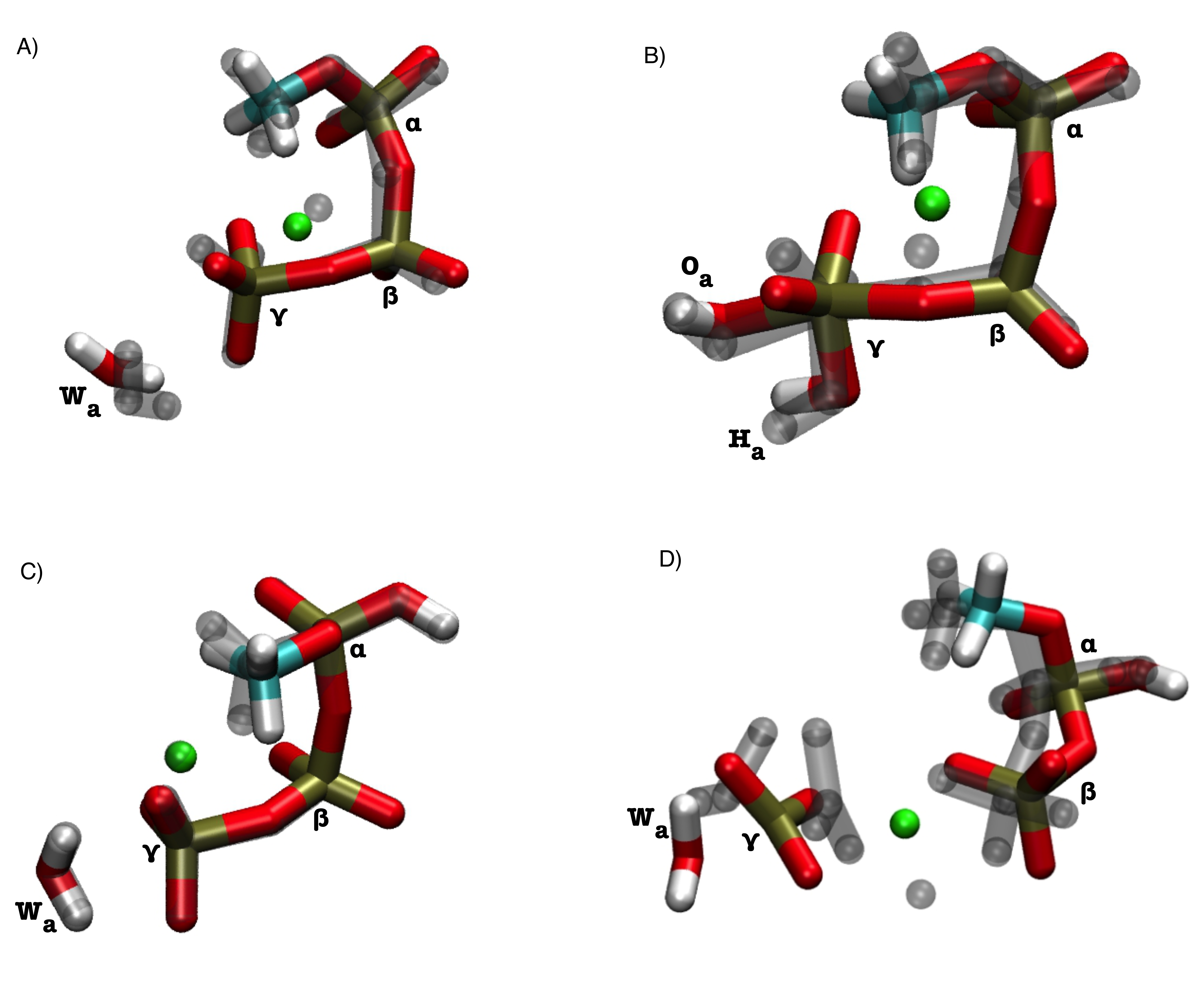


Figure S1: Comparison of structures optimized with B3LYP/6-31+G** (colored) and AM1/d (gray).
**A)** Reactants (as in Figure 1A) and **B)** transition state (energy saddle-point) for the concurrent reaction of fully deprotonated methyl triphosphate. **C)** Reactants and **D)** transition state for the sequential reaction of α-protonated methyl triphosphate. Phosphorus atoms are colored in gold. The four water molecules coordinating the magnesium (in green) are not shown for clarity. The O_a_ label shows the position of the attacking (lytic) water oxygen.

Table S1: Comparison of the AM1/d and B3LYP/6-31+G** methods.[[1]](#footnote-2)

|  | Methyl diphosphate | Methyl triphosphate | |
| --- | --- | --- | --- |
| n_P_ | 0 | 0 | 1 (α) |
| Mechanism | Concurrent | Concurrent | Sequential[[2]](#footnote-3) |
| AM1/d | 35.6 | 44.0 | 30.4 |
| B3LYP/6-31+G** | 40.0 | 42.0 | 33.4 |

Table S2: Modified Phosphorus parameters used in the AM1/d calculations

| USS | -46.58021571 |
| --- | --- |
| UPP | -38.65086942 |
| UDD | -22.82624276 |
| BETAS | -8.00131986 |
| BETAP | -9.18567014 |
| BETAD | -1.34890361 |
| ZS | 2.25736139 |
| ZP | 1.86921196 |
| ZD | 1.53865012 |
| GSP | 6.65536589 |
| HSP | 1.84545119 |
| ALP | 2.00494611 |
| ZSN | 1.68739849 |
| ZPN | 1.26588636 |
| ZDN | 1.06324021 |
| FN11 | -0.06716557 |
| FN21 | 6.17251984 |
| FN31 | 1.22501311 |
| FN12 | -0.00085077 |
| FN22 | 6.26782768 |
| FN32 | 1.82159067 |
| FN13 | -0.06280397 |
| FN23 | 7.46229028 |
| FN33 | 2.38246007 |
| POCORE | 1.30476268 |

**Movies**

Movies C1 to C5 are showing the concurrent reactions, Movies S1 to S7 are showing the sequential reactions. They are in the Mpeg-4 format. Equivalent movies in the Mpeg-1 format can be downloaded from http://www.iwr.uni-heidelberg.de/groups/biocomp/fischer.

Movie C1: Concurrent minimum energy path (MEP) for hydrolysis of unprotonated triphosphate (n_P_ = 0).

Movie C2: Concurrent MEP for hydrolysis of triphosphate singly protonated (n_P_ = 1) on the γ‑phosphate.

Movie C3: Concurrent MEP for hydrolysis of triphosphate doubly protonated (n_P_ = 2) on α and γ‑phosphate.

Movie C4: Concurrent MEP for hydrolysis of triphosphate doubly protonated (n_P_ = 2) on β and γ‑phosphate.

Movie C5: Concurrent MEP for hydrolysis of triphosphate triply protonated (n_P_ = 3) on α,β and γ‑phosphate.

Movie S1: Sequential MEP for hydrolysis of un-protonated triphosphate (n_P_ = 0).

Movie S2: Sequential MEP for hydrolysis of triphosphate singly protonated (n_P_ = 1) on the α‑phosphate.

Movie S3: Sequential MEP for hydrolysis of triphosphate singly protonated (n_P_ = 1) on the β‑phosphate.

Movie S4: Sequential MEP for hydrolysis of triphosphate doubly protonated (n_P_ = 2) on the α,β‑phosphates.

Movie S5: Sequential MEP for hydrolysis of triphosphate doubly protonated (n_P_ = 2) on the α,γ‑phosphates.

Movie S6: Sequential MEP for hydrolysis of triphosphate doubly protonated (n_P_ = 2) on the β,γ‑phosphates.

Movie S7: Sequential MEP for hydrolysis of triphosphate triply protonated (n_P_ = 3) on the α,β,γ‑phosphates.

1. Rate-limiting energy barriers (kcal mol^-1^) of hydrolysis. n_P_ is the number of protons on the phosphates. [↑](#footnote-ref-2)
2. Energy of the transition state for attack of water W_a_ onto the PγO3^-^ metaphosphate moiety (Figure 1D). [↑](#footnote-ref-3)
